# Diabetes mellitus, hearing loss, and therapeutic interventions: A systematic review of insights from preclinical animal models

**DOI:** 10.1371/journal.pone.0305617

**Published:** 2024-07-10

**Authors:** Rahul Mittal, Grant Keith, Mitchel Lacey, Joana R. N. Lemos, Jeenu Mittal, Amro Assayed, Khemraj Hirani

**Affiliations:** 1 Diabetes Research Institute, University of Miami Miller School of Medicine, Miami, Florida, United States of America; 2 Department of Otolaryngology, University of Miami Miller School of Medicine, Miami, Florida, United States of America; 3 School of Medicine and Public Health, University of Wisconsin, Madison, Wisconsin, United States of America; 4 Herbert Wertheim College of Medicine, Florida International University, Miami, Florida, United States of America; Universidad de Chile, CHILE

## Abstract

**Objectives:**

The aim of this systematic review article is to evaluate the relationship between diabetes mellitus (DM) and sensorineural hearing loss (SNHL) utilizing preclinical animal models. The review focused on studies assessing SNHL in diabetic animal models, elucidating the mechanisms of DM-associated SNHL, and exploring the response of diabetic animal models to noise overexposure. We also discussed studies investigating the efficacy of potential therapeutic strategies for amelioration of DM-associated SNHL in the animal models.

**Methods:**

A protocol of this systematic review was designed a priori and was registered in the PROSPERO database (registration number: CRD42023439961). We conducted a comprehensive search on PubMed, Science Direct, Web of Science, Scopus, and EMBASE databases. A minimum of three reviewers independently screened, selected, and extracted data. The risk of bias assessment of eligible studies was conducted using the Systematic Review Center for Laboratory Animal Experimentation (SYRCLE) tool.

**Results:**

Following the screening of 238 studies, twelve original articles were included in this systematic review. The studies revealed that hyperglycemia significantly affects auditory function, with various pathological mechanisms contributing to DM-induced hearing impairment, including cochlear synaptopathy, microangiopathy, neuropathy, oxidative stress, mitochondrial abnormalities, and apoptosis-mediated cell death. Emerging interventions, such as Asiaticoside, Trigonelline, Chlorogenic acid, and Huotanquyu granules, demonstrated efficacy in providing otoprotection for preserving cochlear hair cells and hearing function.

**Conclusions:**

Our systematic review delves into the intricate relationship between DM and hearing impairment in animal models. Future research should focus on targeted therapies to enhance cochlear mitochondrial function, alleviate oxidative stress, and regulate apoptosis. The association between SNHL and social isolation as well as cognitive decline underscores the necessity for innovative therapeutic modalities addressing yet undiscovered mechanisms. Translating findings from animal models to human studies will validate these findings, offering a synergistic approach to effectively manage DM-associated co-morbidities such as hearing impairment.

## Introduction

Diabetes mellitus (DM) is a metabolic disorder characterized by hyperglycemia [1]. In Type 1 diabetes (T1D), hyperglycemia is a result of autoimmune destruction of pancreatic beta cells leading to an absolute deficiency of insulin secretion [2]. In Type 2 diabetes (T2D), hyperglycemia arises from insulin resistance with an inadequate compensatory insulin secretory response [3, 4]. Over the last few decades, the number of people with DM globally has risen from 108 million people in 1980 to 422 million in 2014, with the vast majority being T2D. As of 2021, there are approximately 8.4 million individuals worldwide with T1D, with a modeling study predicting that by 2040 this number could increase to 13.5–17.4 million individuals [5].

To study the pathophysiology of a disease, availability of an animal model that closely recapitulates human clinical conditions is of utmost importance. Before 1987, suitable animal models for understanding the molecular basis of diabetes mellitus (DM) were not available. The landscape changed following the effects of low dose streptozotocin (STZ) on experimental animals. Mice treated with STZ exhibited insulin deficiency, hyperglycemia, polydipsia, and polyuria, mirroring conditions seen in humans with T1D [6]. Subsequent studies have replicated and expanded upon these models [7, 8]. These animal models serve as invaluable tools, enabling researchers to delve into the mechanisms underlying DM, explore complications arising from prolonged hyperglycemia and diabetes, and assess the efficacy of potential therapeutic interventions [9, 10].

**Fig 1:**
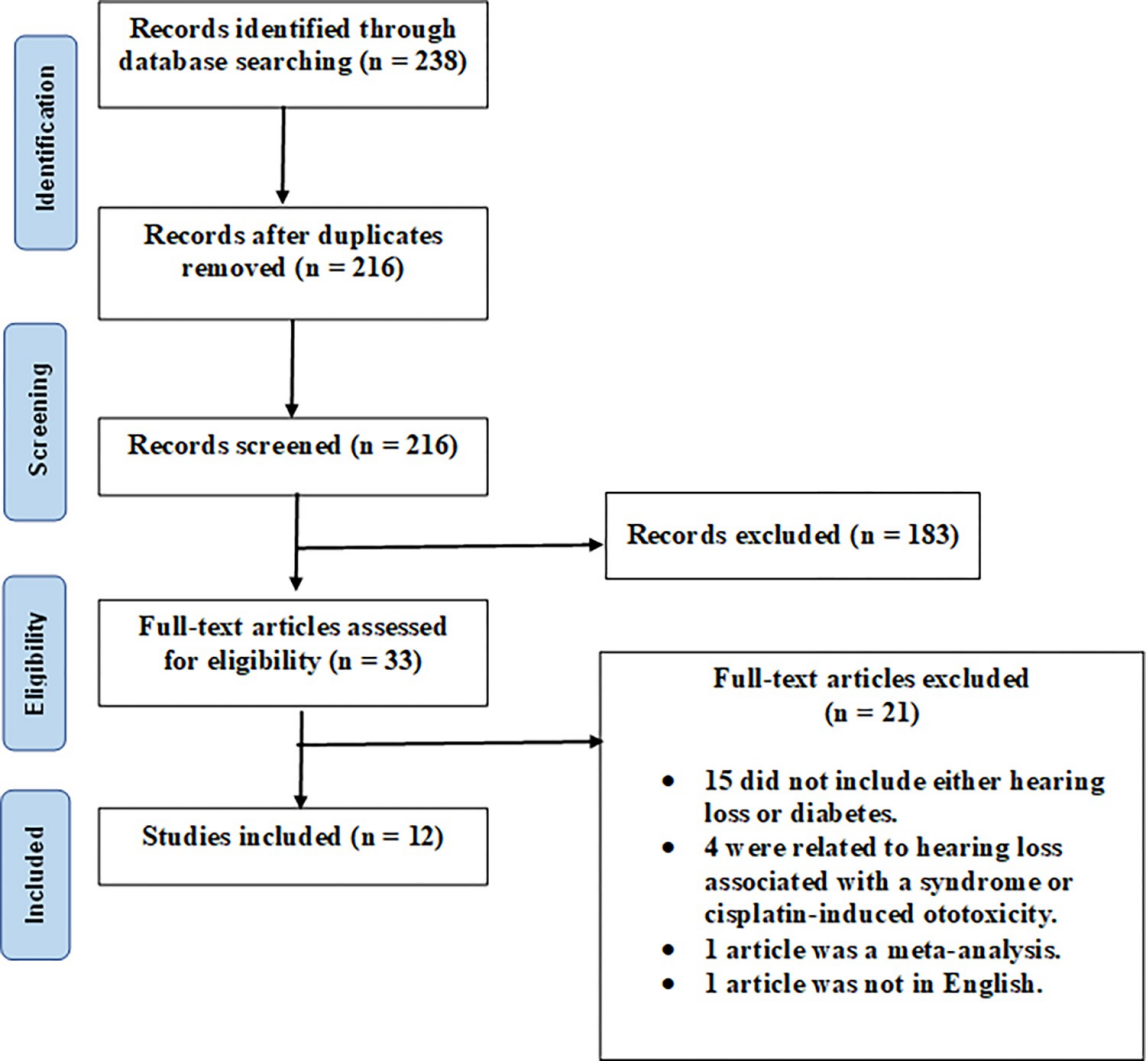
Preferred reporting items for systematic reviews and meta-analyses (PRISMA) flowchart showing the article selection process.

**Fig 2:**
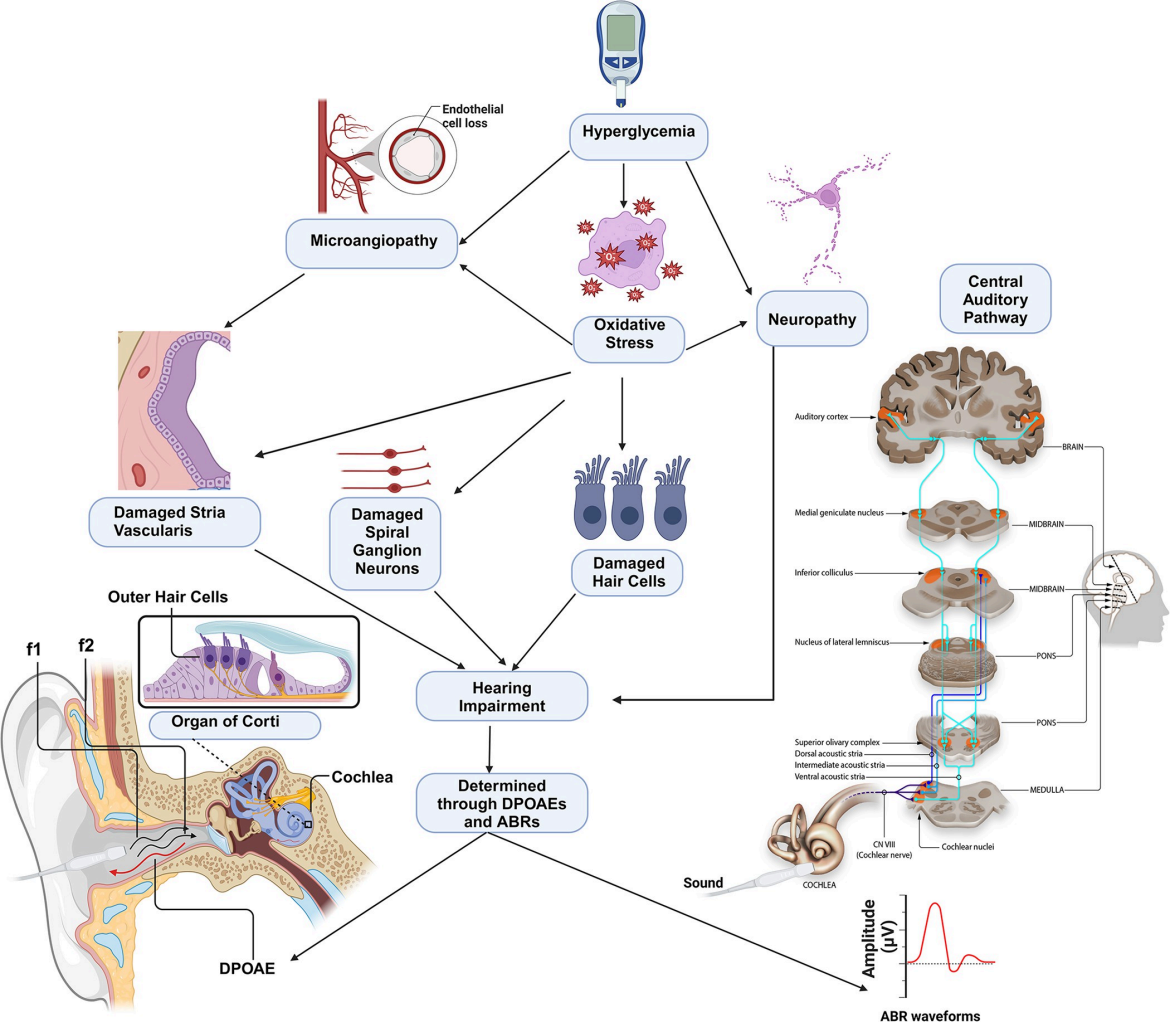
A schematic representation of potential molecular mechanisms underlying diabetes-induced hearing loss. The hyperglycemia prevalent during diabetes initiates a cascade of pathological events, leading to microangiopathy, oxidative stress, and neuropathy. These molecular events can cause damage to the sensory structures such as stria vascularis, spiral ganglion neurons and hair cells leading to hearing impairment. This damage is measurable through two key auditory tests: distortion product otoacoustic emissions (DPOAE) and auditory brainstem responses (ABRs). DPOAE assesses the function of outer hair cells in the cochlea. A probe with two speakers is inserted into the external ear canal to deliver two different frequency tones (f1 and f2). These tones travel through the ear, reaching the cochlear receptor and generating mechanical distortions at various basilar membrane positions, including the prominent 2f1-f2 location. The interaction of these tones depends on the normal function of outer hair cells, which amplify sound through electromotility. The resulting distortions travel back to the external ear and are recorded by a sensitive microphone, measured as DPOAEs in terms of frequency and amplitude in dB SPL. On the other hand, ABRs is an electrophysiological test that evaluates the integrity of the central auditory pathway from the cochlea to the brainstem. Electrodes placed on the scalp capture electrical activity generated by the auditory nerve and brainstem in response to sound stimuli, usually clicks or tone bursts, delivered through earphones. The recorded neural responses form a characteristic waveform, with several peaks corresponding to different points in the central auditory pathway. The central auditory pathway image was taken from Wikimedia Commons uploaded by Jonathan E. Peelle from https://osf.io/u2gxc/ under the Creative Commons Attribution 4.0 International license. Created with BioRender.com.

The long-standing hyperglycemia inherent in DM presents multifaceted challenges, including recurrent life-threatening episodes of both hypoglycemia and ketoacidosis [11]. Furthermore, DM is implicated in various complications, encompassing macrovascular issues such as atherosclerosis and thrombosis in the heart, peripheral arteries, and brain, as well as microvascular complications such as nephropathy, neuropathy, and retinopathy [12–15]. Recent investigations have focused on elucidating the correlation between DM and sensorineural hearing loss (SNHL), unveiling a robust association between DM and auditory impairment in several studies [16–18] (**Fig 1**). The potential molecular mechanisms underlying hearing impairment in diabetes involve hyperglycemia-induced microangiopathy, oxidative stress, and neuropathy. These pathological processes can damage sensory structures in the cochlea, including the stria vascularis, spiral ganglion neurons, and hair cells, ultimately leading to hearing impairment (**Fig 2**).

SNHL can be determined in diabetic animal models utilizing different auditory techniques. Auditory brainstem response (ABR) has long served as a non-invasive tool in audiology clinics for evaluating hearing function [19–24] (**Fig 2**). ABR measures brainstem activity in response to noise exposure and has evolved into a valuable indicator of cochlear synaptopathy in animal models [25–27] (**Fig 2**). The concept of synaptopathy was introduced when studies demonstrated reduced wave 1 of ABR in mice with histologically confirmed synapse loss following noise exposure [28]. Subsequent research in other rodent species [29] and primates [30] supported this concept, while additional studies suggested associations with age [31] and drug ototoxicity [32, 33]. Another frequently used measure in researching SNHL in animal models is distortion-product otoacoustic emissions (DPOAEs), emissions produced by the outer hair cells (OHCs) of the inner ear in response to tones [34–38] (**Fig 2**). Reduction or absence of these emissions indicates functional disorders of the inner ear, such as damage to the OHCs [39–41].

The objective of this systematic review article is to discuss the recent advancements in understanding the correlation between DM and SNHL utilizing preclinical animal models. We discussed studies that assessed the extent of SNHL in diabetic animal models as well as underlying molecular mechanisms. We also focused on studies that deciphered the effect of noise overexposure as well as determined the efficacy of novel treatment modalities for hearing loss in preclinical diabetic animal models.

## Methods

### Search strategy

This study was conducted following the Preferred Reporting Items for Systematic Reviews and Meta-Analyses (PRISMA) statement and supplemented by guidance from the Cochrane Collaboration Handbook. A protocol of this systematic review was designed a priori and was registered in the PROSPERO database (registration number: CRD42023439961). As narrative or systematic review articles were available covering studies up to 2016, searches were performed from January 1, 2017 until June 6, 2023 in the following databases: PubMed (MEDLINE), Science Direct, Web of Science, Scopus, and EMBASE using the following MeSH terms: “Diabetes Mellitus”[Mesh] AND “Hearing Loss”[Mesh] AND “Therapeutic Interventions” [Mesh] AND (“Mice”[Mesh] OR “Rat”[Mesh]. Where MeSH search was not available the following Boolean terms were used “Diabetes Mellitus” AND “Hearing Loss” AND “Therapeutic Interventions” AND (“Mice” OR “Rat”).

### Study selection

Studies were excluded based on the following exclusion criteria: studies that did not fit the above characteristics, review articles, meta-analyses, abstracts only, conference proceedings, editorials / letters, case reports, or articles published before 2017. Additionally, publications not in English language were not included in this search. The articles before 2017 were not included as there was a previous review covering the studies up to 2016 regarding DM-induced SNHL in animal models. The following inclusion criteria were used: experimental studies focused on hearing loss in diabetic mice or rat models, including the extent of hearing loss, the mechanisms behind hearing loss in the setting of diabetes, and therapies targeted at DM-associated hearing loss in animal models. All searched titles, abstracts, and full-text articles were independently reviewed by at least two reviewers (G.K., M.L, K.M., and R.M.). Disagreements over inclusion and exclusion criteria were resolved through a consensus between the reviewers or discussion with other investigators of this study. Articles were initially screened based on title and abstract screening before proceeding to full-text analysis.

### Data extraction

All data were extracted by at least two trained reviewers (G.K., M.L., K.M., and R.M.). The investigations that were performed to evaluate the effect of diabetes on the auditory system were grouped together and those that were evaluating possible therapies were grouped together.

### Quality assessment

The risk of bias was assessed using the Systematic Review Center for Laboratory Animal Experimentation (SYRCLE) tool [42–48]. This tool adapts concepts from the Cochrane Collaboration’s tool for assessing the risk of bias in clinical trials, tailoring them to address the unique aspects of animal experimentation. It encompasses several domains, including selection bias, performance bias, detection bias, attrition bias, and reporting bias. Each domain contains specific criteria that help to assess potential biases in study design, conduct, and reporting. Each study was graded to either be of “low,” “high,” or “unclear” risk. At least two reviewers independently conducted this assessment (G.K., M.L., K.M., R.M.), and any disagreements were resolved by consensus between the reviewers or discussion with other investigators of this study.

## Results

A total of 238 studies were identified using the search terms described in the methods section. After eliminating 22 duplicate records, 216 studies underwent title and abstract screening. Following this screening process, 183 studies were excluded based on irrelevance, primarily pertaining to hearing loss associated with a syndrome other than DM. Ultimately, 33 articles proceeded to full-text analysis. Within this subset, 21 articles were excluded, with 15 not addressing SNHL or DM, 4 focusing on hearing loss related to a syndrome other than DM, one being a meta-analysis, and one not published in English. The final selection for this systematic review comprised 12 articles published between 2017 and 2023. The search strategy, along with the study inclusion process, is depicted in the PRISMA diagram in Fig 1.

**Fig 3:**
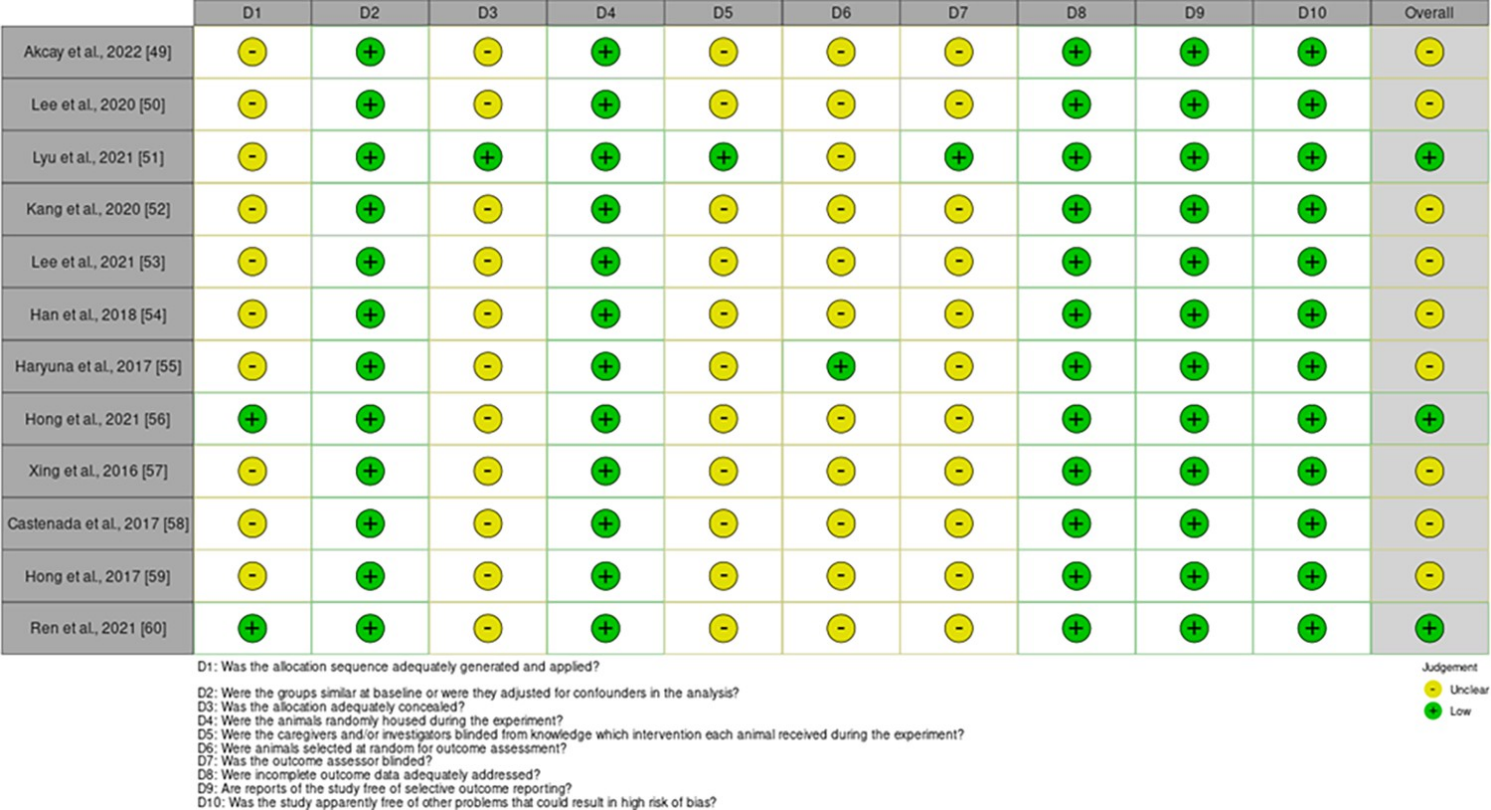
Assessment of risk of bias using the Systematic Review Center for Laboratory Animal Experimentation (SYRCLE) tool.

The risk of bias analysis is shown in Fig 3. A summary of studies included in this systematic review is presented in Table 1.

**Table 1:** A summary of studies included in this systematic review.

Reference	Study	Animal species/strain	Exposure	Comparison	Outcomes
Akcay et al., 2022 [49]	Quasi-experimental study	25 female Wistar rats: • 7 HBG • 8 DM • 10 controls	Streptozotocin induced DM	Non-diabetic controls	• Significant difference in threshold values at 8kHz and 16kHz between DM and control groups. • Significant differences in interpeak latencies at 8kHz and 16kHz between DM and control groups.
Lee et al., 2020 [50]	Quasi-experimental study	16 mice: • 8 Type 1DM Akita • 8 WT controls	Akita Type 1 Diabetes	Non-diabetic WT controls	• Significant increase in ABR thresholds at 16 and 32 kHz compared to WT group. • Significant decrease in amount of spinal ganglion neurons compared to WT group. • No change in IHCs, OHCs, and supporting cells. • Significant decrease in thickness of the stria vascularis in the middle and basal turns of the cochlea compared to WT group.
Lyu et al., 2021 [51]	Quasi-experimental study	42 male mice: • 22 diabetic Lepr db/db • 20 db/+ non-diabetic control	T1D	Non-diabetic controls	• Significant increase in hearing thresholds at 32 kHz compared to control group as early as 6 weeks of age. • No significant difference regarding IHC and OHC survival in the apex and middle turns of the cochlea, but significant decrease in hair cell survival at the basal turn of the diabetic mice. • Significant reduction in the number of synaptic ribbons on IHCs in the diabetic group.
Kang et al., 2020 [52]	Quasi-experimental study	8 week old C57BL/6 mice, unclear sample size.	Streptozotocin induced DM	Non-diabetic controls	• Increased damage to the cochlear afferent nerve fibers in hyperglycemic group. • Decreased proportion of calretinin-poor cochlear afferent fibers compared to normoglycemic group.
Lee et al., 2021 [53]	Quasi-experimental study	20 male Sprague-Dawley rats, 8 weeks of age: • 4 short term control • 4 long term control • 6 short term DM • 6 long term DM	Streptozotocin induced DM	Non-diabetic controls	• Control group hearing thresholds returned to baseline following noise exposure, whereas DM group hearing thresholds did not recover at 12, 16, and 32 kHz. • DM groups had larger peak 1 amplitudes at low frequencies at baseline, which persisted in the 14 days post-noise exposure.
Han et al., 2018 [54]	Quasi-experimental study	60 7 week old mice: • 30 db/db diabetic • 30 WT	Noise Exposure	No noise exposure	• Significantly greater ABR threshold shifts after noise trauma in the diabetic group. • Complete loss of OHCs following noise trauma in both diabetic and WT groups. • Significantly more preservation of IHCs after noise trauma in WT group.
Haryuna et al., 2017 [55]	Quasi-experimental study	24 Wistar rats: • 4 in control group • 4 in 4 tx groups	Dose Dependent Curcumin Tx	Non-diabetic controls	• Significant decrease in Type IV collagen expression in the cochlear lateral wall in the DM group compared to the control group. • Significant increase in Type IV collagen expression in the cochlear lateral wall in the long term Curcumin treatment groups compared to the non-treatment diabetic group.
Hong et al., 2021 [56]	Quasi-experimental study	60 SPF SD rats: • 10 non-diabetic controls • 10 diabetic controls • 10 high dose Huotanquyu tx • 10 medium dose Huotanquyu tx • 10 low dose Huotanquyu tx • 10 mecobalamin tx	Dose Dependent Huotanquyu Tx	Both diabetic and non-diabetic untreated control groups	• Statistically significant increase in the counts of spiral ganglion cells in the cochlear fundus gyrus of diabetic rats in the low, medium, and high dose Huotanquyu groups when compared to the diabetic model control group. • Reduced thickening of the basement membranes and stria vascularis and more dense helical ganglion cells in the diabetic Huotanquyu treatment groups compared to the diabetic model control group. • Changes in hair cells on electron microscopy among the control group, the diabetic untreated group, and Huotanquyu treatment groups.
Xing et al., 2016 [57]	Quasi-experimental study	BALB/c 4 week old mice, unclear sample size: • DM control group • DM 5mg/kg Asiaticoside tx • DM 15mg/kg Asiaticoside tx • DM 45mg/kg Asiaticoside tx • Non-diabetic, non-treated control group	Dose Dependent Asiaticoside Tx	Both diabetic and non-diabetic untreated control groups	• Significantly increased hearing thresholds at 4, 8, 16, and 32 kHz for the Streptozotocin induced DM control group compared to the non-diabetic control group. • Significantly decreased number of IHCs and OHCs in the DM control group compared to the non-diabetic control group. • Significantly higher number of IHCs and OHCs in the high dose Asiaticoside diabetic tx group compared to the DM untreated group. • Significantly decreased AGE/RAGE/Nf-Kb among the Asiaticoside treatment groups compared to the DM untreated group.
Castaneda et al., 2017 [58]	Quasi-experimental study	40 eight-week-old male LepR mice: • 10 db/db DM group • 10 db/ + non-diabetic group • 10 db / db mice treated with 10 mg/kg Trigonelline • 10 db / db mice treated with 20 mg/kg Trigonelline	Dose Dependent Trigonelline Tx	Both diabetic and non-diabetic untreated control groups	• Hearing thresholds for ABRs were higher in the DM untreated group than the non-diabetic control group. • Hearing thresholds were significantly decreased in both the TRG treatment groups compared to the untreated DM group. • Cochlear response by SNR for TEOAE was increased in both the TRG treatment groups compared to the untreated DM group.
Hong et al., 2017 [59]	Quasi-experimental study	40 eight week old male mice: • 10 non-diabetic controls • 10 db/db DM • 10 DM treated with 10 mg/kg Chlorogenic Acid • 10 DM treated with 20 mg/kg Chlorogenic Acid	Dose Dependent Chlorogenic Acid Tx	Both diabetic and non-diabetic control groups	• Significant increase in hearing thresholds at 4 kHz and 8 kHz in the DM untreated group compared to the non-diabetic control group. • Significant decrease in hearing thresholds at same frequencies in the DM groups treated with Chlorogenic Acid compared to the DM untreated group. • Morphologically, the OHCs in mice treated with Chlorogenic acid were more similar to the non-diabetic control group than the untreated DM group.
Ren et al., 2021 [60]	Quasi-experimental study	Six week old male C57BL/6J mice, unclear sample size	Upregulation of Trx by treatment with SF	Both diabetic and non-diabetic control groups	• Significant increase in the rates of OHC and IHC loss in the diabetic group compared to the non-diabetic control group. • Significant reduction in rates of OHC and IHC loss in the Trx upregulation diabetic group compared to the untreated diabetic group. • Significant downregulation of Trx and downregulation of ASK1 expression in the diabetic group compared to the WT group.

### Blood glucose levels and SNHL

While there is a relatively well-understood relationship between hyperglycemia and microvascular damage to peripheral nerves, retina, and the kidneys [12], there is much less information that specifically looks into other complications of the long-standing hyperglycemia in DM such as hearing function. Akcay et al. aimed to evaluate whether changes in blood glucose levels would impact hearing function measured via brainstem auditory evoked potentials (BAEP) and DPOAEs in a streptozotocin induced rat model of DM [49]. Three months later, blood samples were collected from the animals to measure their blood glucose levels. Rats with blood glucose between 100 and 300 mg/dL were grouped into the high blood glucose (HBG) group while rats with blood glucose levels over 300 mg/dL were categorized into the DM group. One month later fasting blood glucose levels were taken again followed by BAEP and DPOAE recordings. The DM group showed significantly higher blood glucose levels of 421.50 +/- 35.93 mg/dL compared to HBG and control groups having glucose levels of 213.83 ± 29.18 (p<0.05) and 104.33 ± 3.45 (p<0.01) mg/dL, respectively. The BAEP recordings revealed that at 8 kHz, the threshold values increased only in the DM group compared to the control group. At 16 kHz, the threshold values were higher in both the HBG and DM groups than the control group (p<0.05). In ABR evaluation, I, II, III, IV, and V wave latencies of the DM group were significantly higher than the control group at both 8 kHz (p<0.01) and 16 kHz (p<0.05). However, I, II, III, and IV wave latencies of the HBG group were higher than the control group only at 8 kHz (p<0.05). Although there was a prolonged latency of BAEP compared to the control group at 16 kHz, the elongation was not statistically significant. There were significant differences between the DM and control groups’ wave I-III, III-V, and I-V interpeak latencies at 8 kHz (p<0.05) and 16 kHz (p<0.05), but no differences were observed between the HBG and the control groups’ interpeak latencies. Overall, there was a positive correlation between blood glucose level and I, II, III, IV, and V waves latencies and threshold values at 8 kHz. There was also a positive correlation between blood glucose level and latency of III wave only at 16 kHz. The DPOAE values revealed significant decreases in the signal to noise ratio (SNR) values at 4000, 6000, 8000, and 10000 Hz in the DM group compared to the control group, whereas the SNR values in the HBG group were only decreased compared to the control group at 6000, 8000, and 10000 Hz. These results suggest damage to the cochlea and auditory pathways occurs not only in DM but also with high blood glucose levels.

### DM induced SNHL: Molecular, functional, and histological characterization

In order to completely understand the impact of DM on SNHL, it is imperative to evaluate the anatomical changes that occur in the ear with DM. A study by Lee et al. determined the correlation between T1D and SNHL through analysis of both hearing function and immunohistochemical changes in diabetic Akita mice and comparing it with wild-type (WT) mice [50]. The Akita diabetic mouse model group had significantly elevated glucose levels and insulin sensitivity compared to the WT group initially. At 3 months, the Akita group had significantly increased ABR thresholds at 16 and 32 kHz compared to the WT group, supporting the concept that T1D causes hearing impairment.

During immunohistochemical analysis of the cochleae, the Akita mouse group had a significant decrease in the amount of spinal ganglion neurons compared to the WT group. Further microscopic evaluation revealed abnormal mitochondria, which resulted in disruption of the normal structures of the spinal ganglion neurons. However, the inner hair cells (IHCs), outer hair cells (OHCs), and supporting cells (SCs) showed no significant changes between the Akita group and the WT group. The stria vascularis (SV) in the Akita group had significantly decreased thickness in the middle and basal turns of the cochlea, with a particular decrease in intermediate cell density and a loss of CD31 expression in the vessel walls. Additionally, a reduction in type I, II, and IV fibrocytes and Na^+^/K^+^ ATPase α1 expression in spiral ganglion neurons was observed. These results suggest that the hearing loss associated with T1D is associated not only with ion imbalances and blood flow disorders of the cochlear endolymph, but also with the degeneration of the cochlear sensory structures via apoptosis-mediated cell death.

Lyu et al. aimed to further assess the pathophysiology of DM associated hearing impairment and cochlear synaptopathy using B6.BKS(D)-*Lepr*^db^ diabetic mouse model [51]. Hearing thresholds were determined by ABRs from 4 weeks of age to 14 weeks of age followed by cochlear evaluation. The diabetic mice had significantly increased hearing thresholds at 32 kHz compared to the control group mice as early as 6 weeks of age, as well as increased thresholds in all other frequencies at later time points, confirming that the diabetic group had significant hearing impairment compared to the control group. There was no significant difference regarding both inner and outer hair cell (HC) survival in the apex and middle turns of the cochlea between the two groups, but there was a significant decrease in HCs in the basal turn of the diabetic mice compared to the control group. However, this did not appear to be a sufficient explanation for the hearing impairment, so the cochlear synapses were evaluated. The cochleae of the diabetic mice group expressed a significant reduction in the number of synaptic ribbons on IHCs. Analysis of the ABR wave-I amplitude revealed significant decreases (p<0.05) in wave-I amplitude among the diabetic mice group at all levels (60–90 dB) at 6, 10, and 14 weeks old, which is consistent with the cochlear synapse results. These results support the idea that cochlear synaptopathy contributes more to diabetic hearing impairment than HC does.

Further histopathological analysis focused on the mitochondria of IHCs, OHCs, and synapses revealed that the diabetic mice group had significant mitochondrial abnormalities compared to the control group, including vacuolated mitochondria with disrupted cristae. Analysis of SV revealed similar results, specifically in the intermediate cells. Assessment of the underlying mechanisms behind the mitochondrial damage in the diabetic mice via quantitative reverse-transcription polymerase chain reaction (qRT-PCR) and Western blotting analysis suggested that the cochleae of the diabetic mice group had increased levels of pro-inflammatory cytokines, impaired mitochondrial biogenesis, and impaired glucose and fatty acid metabolism in comparison to the cochleae of the control group mice. Protein analysis of the cochleae revealed that there were significantly decreased levels of BCL-2 and caspase-9 in the diabetic group compared to the control group, indicating mitochondria-mediated apoptotic cell death in the diabetic mice cochleae. However, there were no changes revealed among necroptotic programmed cell death markers between the diabetic and control groups. Overall, these results indicate that DM-induced HL is associated with synaptopathy, microangiopathy, mitochondrial structural disruption / function impairment, and activation of the intrinsic apoptosis pathway. The researchers urge future studies to investigate therapeutic strategies for DM associated HL that specifically target mitochondria.

Another study assessed whether cochlear afferent fibers that are deficient in the calcium buffering protein calretinin are more vulnerable to hyperglycemic insults using streptozotocin induced mouse model of DM [52]. Initial analysis of both normoglycemic and hyperglycemic cochleae supported recent studies findings that calretinin-poor cochlear afferent fibers tend to contact the modiolar side of inner hair cell membranes while calretinin-rich cochlear afferent fibers contact all sides of the inner hair cell. Further analysis revealed that the hyperglycemic group of mice had increased damage to their cochlear afferent nerve fibers and a decreased proportion of calretinin-poor cochlear afferent fibers compared to the normoglycemic group of mice. These results suggest that the calretinin-poor cochlear nerve fibers may selectively be lost following the hyperglycemic insults and indicate that these fibers may play a protective role against hyperglycemic insults.

### Diabetic animal models and noise overexposure

Studies have shown that noise overexposure further deteriorates the complication of HL due to DM and hyperglycemia. Lee et al. evaluated changes in hearing function and synaptic changes in the IHCs of rats with streptozotocin induced DM following noise overexposure [53]. Animals were divided into four groups: control short-term (CST), control long-term (CLT), DM short-term (DMST), and DM long-term (DMLT). The rats that were in the short-term groups were exposed to noise for 4 weeks, whereas animals in the long-term groups were exposed to noise for 12 weeks following streptozotocin administration. This noise was narrow-band, centered at 16 kHz. ABRs were measured at baseline, before, and at 1 and 14 days after noise exposure followed by harvesting of cochleae. Results confirmed that the streptozotocin administration led to hyperglycemia confirming establishment of a diabetic rat model in the DMST and DMLT groups. Prior to noise exposure, the hearing thresholds of the control and DM groups were similar. Hearing was then monitored for 14 days after noise exposure, which revealed initial increases in the threshold shifts in all groups. However, in the CST and CLT groups, the hearing thresholds returned to normal at 14 days, whereas there was no recovery in the DMST and DMLT groups. These results suggest noise exposure can produce permanent hearing threshold shifts in diabetic animals.

Additionally, researchers analyzed peak 1 amplitude at baseline and 14 days post-noise exposure in order to assess the signaling intensity of the cochlea. Results revealed increases (p<0.01) in peak 1 amplitude at low frequencies (4 kHz and 8 kHz) in both of the diabetic rat groups compared to the control groups. At both baseline and 14 days post-noise exposure, the peak 1 amplitude was significantly higher at 4 and 8 kHz in the diabetic groups than the control groups. Alternatively, the baseline peak 1 amplitudes were not significantly different among groups at the higher frequencies. 14 days post-noise exposure, the peak 1 amplitudes were significantly larger in the DMST and DMLT groups than the CST and CLT groups. The larger peak 1 amplitudes at low frequencies seen in the diabetic groups at baseline persisted in the 14 days post-noise exposure measurements, and the peak 1 amplitudes at the higher frequencies increased in both diabetic groups at that time. These findings suggested that the noise-induced decrease in the peak 1 amplitude at the high frequencies observed in control animals was absent in the diabetic groups, potentially indicating that the synaptopathy was less severe in the diabetic animals. To further confirm this functional change, they evaluated the distribution and density of presynaptic puncta on IHCs of the cochleae. While there was no observed loss of IHCs and the distribution of the presynaptic puncta was similar among all the groups, synaptic damage differed such that the DMLT group had significantly more synapses in the 16 kHz region than the DMST and control groups. This finding further supports the concept that noise exposure produced less damage to the ribbon synapses in the diabetic animals than in the control animals.

Another study by Han et al. determined whether diabetic mice (db/db) are more susceptible to noise trauma (NT) than WT animals using ABRs followed by cochlear morphological studies and determination of inflammatory responses [54]. To induce NT, both diabetic and WT mice were exposed to broadband noise (250 to 8 kHz) at 116 dB SPL for 1 h. ABR threshold shifts were significantly greater at 1 and 2 weeks after NT in db/db diabetic mice compared to WT group (p<0.05). These results suggest that db/db mice cochleae were more susceptible to NT-induced damage compared to WT animals. Immunohistochemistry performed two weeks after NT revealed almost complete loss of OHCs in both diabetic and WT mice in the basal turn of the cochlea. However, IHCs were more preserved in WT mice compared to diabetic group (p< 0.05). In the middle turn, greater preservation of the outer hair cells was observed in WT mice compared with db/db mice (p< 0.05). The number of surviving hair cells was also significantly higher in WT mice (p< 0.05). These findings suggest that the auditory hair cells in db/db diabetic mice are more susceptible to damage in response to NT. In addition, synapse loss in the middle turn of the cochlea was more severe in db/db diabetic mice compared to the WT group, suggesting that in surviving IHCs the synaptopathies were more severe in db/db diabetic group compared to WT animals (p< 0.05). As NT induces oxidative stress, therefore the levels of antioxidant enzymes and reactive oxygen species (ROS) markers such as Heme oxygenase-1 (HO-1), catalase, superoxide dismutase 1 (SOD1) and nuclear respiratory factor 1 (NRF1) were determined using (qRT-PCR). There was an increase in HO-1 and catalase levels at 3 and 7 days whereas SOD1 and NRF1 levels were increased at 7 days in db/db diabetic mice compared to WT animals (p< 0.05). These results suggest that ROS production after NT was significantly higher in the db/db diabetic group compared to WT mice.

NT induces inflammation in the cochlea. Therefore, the expression of interleukin-1β, interleukin-6, nitric oxide synthase 2, tumor necrosis factor-α (TNF-α), and cyclooxygenase 2 (COX2) was determined using qRT-PCR as indicators of the inflammatory response. The levels of IL-1β, IL-6, and TNF-α levels were significantly increased at 3 and 7 days, NOS2 levels at 1 and 3 days, and COX2 levels at 1, 3, and 7 days after NT in db/db diabetic mice compared with WT group (p< 0.05). These results suggested that inflammatory responses in the cochlea were more severe in diabetic mice than in the WT animals. The results of the study suggest that diabetic mice were more susceptible to NT compared to WT mice. Increased inflammatory response and ROS production in diabetic mice can cause auditory HC damage and synaptopathy leading to HL. Further studies are warranted to determine how diabetes makes cochlea more prone to damage in response to NT.

In summary, SNHL associated with diabetes is primarily driven by hyperglycemia-induced mechanisms, including microangiopathy, oxidative stress, and neuropathy causing damage to sensory structures in the cochlea. Therapeutic strategies targeting these underlying mechanisms hold a great potential to prevent DM-induced SNHL. In the following section, we will discuss some of these emerging therapies and determination of their efficacy for DM-induced SNHL using preclinical animal models.

### Using animal models to test novel therapeutics for DM-induced SNHL

Animal models have served as a valuable tool in evaluating the impact of emerging therapeutics targeted specifically at DM induced SNHL. Studies discussed in this section revealed exciting results that will pave the way for developing effective interventions for DM associated SNHL.

#### Curcumin therapy

A study by Haryuna et al. determined whether curcumin is safe and effective in treating damage to fibroblasts in the cochlear lateral wall (LW), which has been suggested as one of the causes of DM associated HL [55]. The rats were divided into six groups, with group 1 being a control group and the remaining five groups were given streptozotocin to initiate pancreatic beta-cell destruction, simulating diabetes. Group 2 served as the DM control group, while groups 3 and 5 received 200 mg/kg per day of curcumin and groups 4 and 6 received 400 mg/kg per day of curcumin. Groups 1–4 were euthanized on the fifth day while Groups 5 and 6 were terminated on the tenth day, with subsequent tissue fixation and analysis of expression of type IV collagen. There was a significant decrease in type IV collagen expression in the cochlear LW in the DM control group (group 2) compared to the control group (group 1) (p = 0.011). The levels of type IV collagen were higher in groups 3 and 4 compared to group 2. However, this increase did not reach a statistically significant level. On the contrary, the type IV collagen expression in cochlear LW in animals in groups 5 and 6 that received curcumin for 8 days showed a statistically significant increase compared to the diabetic rats that did not receive any curcumin (p = 0.010 and p = 0.015, respectively). These results suggest that curcumin could be a viable therapy that can be used in the management of diabetes associated HL by preventing damage to fibroblasts in the cochlear LW.

#### Effect of Huotanquyu granule on cochlear morphology

Hong et al. determined whether the Chinese medicine, Huotanquyu granules, can attenuate cochlear pathology in response DM using a streptozotocin-induced diabetic rat model [56]. Animals received high (31.1 g/kg), medium (15.6 g/kg), and low dose (7.8 g/kg) of Huotanquyu granule through oral gave (savage). Animals receiving saline or the Western medicine mecobalamin (8 mg/kg) served as control and positive groups respectively. The administration of medication was daily for 10 weeks. At the end of week 12, rat cochlear tissues were harvested and analyzed. There was a statistically significant increase in the counts of spiral ganglion cells in the cochlear fundus gyrus of rats in the low, medium, and high dose groups when compared to the diabetic model control group (p<0.05). When looking at the histology of the cochlea, it was observed that the Huotanquyu granule groups had reduced thickening of the basement membranes and stria vascularis, as well as more dense helical ganglion cells compared to the diabetic model group. Additionally, when looking at the hair cells via electron microscopy, researchers found differences between the groups. In the control group, the static cilia of three rows of OHCs and one row of IHCs were arranged in a V shape, with no disorder, deletion, prostrate, or fusion. In the diabetic model group, the static cilia of the three rows of OHCs were different in length, disorderly in arrangement, and had obvious lying down and fusion without obvious loss, while the IHCs were different in length with obviously lying down and shedding. In comparison to the diabetic model group, the high dose and medium dose treatment groups’ static cilia of the three rows of OHCs were arranged in a V shape with significantly reduced lying down and fusion, while the IHCs were arranged in a basic way with no obvious lying down or falling out. The static cilia of the three rows of OHCs in the low dose treatment group were arranged more clearly and had some improvement in prolonging and fusion, but the static cilia of the IHCs still showed prolonging and shedding. These results suggest that the Huotanquyu granules may be beneficial in preventing cochlear damage related to hyperglycemia and DM.

#### Asiaticoside protects cochlear hair cells from high glucose-induced oxidative stress

A study by Xing et al. evaluated the effect of the antioxidant, Asiaticoside, on cochlear hair cells and HL in streptozotocin-induced diabetic mouse models [57]. Animals received 5mg/kg, 15mg/kg, and 45mg/kg of Asiaticoside for 3 weeks. Following treatment, hearing thresholds were determined using ABRs followed by cochlear analysis. Results revealed that the streptozotocin-induced diabetic mouse model had significantly increased hearing thresholds at 4, 8, 16, and 32 kHz. Treatment with Asiaticoside significantly decreased the hearing threshold when compared to the diabetic model group in a dose dependent manner, although. A similar trend was seen regarding the number of IHCs and OHCs, as the diabetic model group had significantly decreased numbers compared to the control group (p<0.05), but the Asiaticoside treated groups had significantly protected hair cell numbers, also in a dose dependent manner (p<0.05 at 15 and 45 mg/kg of AC for OHCs, at all doses for IHCs). Additionally, ELISA testing revealed decreases in AGE/RAGE/Nf-Kb among the animals treated with AC in a dose dependent manner (p<0.05 at all doses). These results indicate that Asiaticoside may provide otoprotection for loss of OHCs and IHCs promoting preservation of hearing function in the setting of hyperglycemia or diabetes, potentially via a mechanism that increases the activity of antioxidants and inhibits the AGEs/RAGE/Nf-Kb pathway.

#### Trigonelline provides otoprotection through nerve growth factor signaling

Castaneda et al. set out to evaluate whether Trigonelline (TRG) would reduce DM-induced auditory damage using LepR(db/db) diabetic mouse model [58]. ABRs to clicks and tone burst stimuli at 4 kHz were measured at the beginning of the study and again after the 8 weeks of treatment with TRG at doses of 10 mg/kg or 20mg/kg. The results of the study revealed that hearing thresholds of both the click and the 4 kHz were higher after 8 weeks in the diabetic mice compared to the non-diabetic control group (p<0.05). Treatment with TRG at both 10 and 20 mg/kg significantly decreased hearing thresholds compared to the non-treated diabetic group (p<0.05) in both click and 4 kHz. Additionally, the results revealed increased cochlear response by SNR for transient evoked otoacoustic emission (TEOAE) in response to 2 kHz and 3 kHz TBs in both of the treatment groups in comparison to the diabetic group (p<0.001). In a separate part of the study utilizing zebrafish as a model to evaluate NGF interaction with neuromast function and otic hair cells, the results supported the notion that TRG action is related to increased nerve growth factor (NGF) (p<0.01 at both 2.5 and 5 micrograms/mL), increased neuromast function, and an increased number of otic hair cells. These results indicate that TRG action on the auditory system during DM-induced HL may be related to a direct interaction with NGF and increased NGF expression.

#### Chlorogenic acid rescue’s sensorineural auditory function

A study by Hong et al investigated the effects of chlorogenic acid (CA) on diabetic auditory pathway impairment utilizing neuro-electrical physiological measurements and morphological investigations [59]. Animals were divided into four groups: a non-diabetic control group (*db/h*), a non-treated diabetic control group (*db/db*), and diabetic groups treated with 10mg/kg and 20mg/kg of CA daily for 8 weeks. Body weights and blood glucose levels as well as ABR, TEOAE, and auditory middle latency responses (AMLR) were determined after 8-weeks of treatment. The diabetic control group exhibited significantly increased body weights and blood glucose levels compared to the non-diabetic control group. However, treatment with 20mg/kg CA significantly decreased body weights compared to the diabetic control group. In addition, the diabetic mice treated with both the 10mg/kg and 20mg/kg doses of CA had significantly decreased blood sugar levels than the untreated diabetic animals.

ABRs revealed significantly higher hearing thresholds in response to clicks, 4 kHz, and 8 kHz TBs in the untreated diabetic group compared to the normal group, whereas the thresholds of the diabetic mice treated with CA were significantly decreased compared to the diabetic control group. To further confirm these findings, AMLR amplitudes and latencies were measured. Pa latencies and Non-Pa amplitudes of the AMLRs in the diabetic control mice were significantly delayed and lower compared to the non-diabetic control group. However, the diabetic mice in the treatment groups had decreased Pa latencies and increased Non-Pa amplitudes compared to the untreated diabetic mice.

Further evaluation of the cochleae with TEOAE SNRs of the diabetic control mice were significantly lower than the non-diabetic control group. Analysis of the hair cells in the organ of Corti using SEM were similar to the physiological findings, as damage to the stereocilia of OHCs occurred in the diabetic untreated mice. In the diabetic mice that received CA, TEOAE SNRs were increased significantly in a dose dependent manner excluding 4 kHz TB stimulus. Additionally, the morphologic results of OHCs in the diabetic mice treated with CA were similar to the non-diabetic control mice.

These results suggest that CA may improve hyperglycemia and improve damaged peripheral auditory function in diabetic mouse models. The AMLR results provide support for the idea that the central auditory pathway may become affected in diabetic mice over time, whereas CA may suppress central auditory pathway dysfunction in diabetic mouse models. The results of the cochlear morphologic analyses suggest that CA may aid in the recovery of OHC damage in the cochlea.

#### Upregulation of thioredoxin provides protection against DM-induced hearing impairment

A study by Ren et al identified the protective effect of Thioredoxin (Trx) on DM-induced SNHL and to identify an early potential therapeutic target for diabetic hearing impairment in the future [60]. Thioredoxin (Trx) is a small redox protein present in tissues which contains dithiol-disulfide active site, which functions to regulate oxidative stress [61], endoplasmic reticulum stress [62], and autophagy in biological processes [63]. When oxidized Trx is a vital regulator of apoptotic signal-regulating kinase 1 (ASK1) by dissociating from ASK1 and interacting with TRAF2/6 forming an apoptotic complex promoting cell apoptosis and death [64]. Additionally, Trx regulates autophagy through thioredoxin-interacting protein (Txnip)-mTOR pathway [63]. Trx transgenic (Tg) mice were injected with streptozotocin and with/without sulforaphane (SF) or PX12 treatment and evaluation of HC loss was performed. As diabetes progressed, insulin resistance index increased in diabetic group compared to the control group. Diabetic mice were observed to have degeneration of OHCs and IHCs. The degeneration started at the basal turn and expanded toward the apical turn. The percentage of loss increased with duration of diabetes, although IHC degeneration was slower than OHC degeneration. Trx expression gradually decreased and was positively correlated with the loss of cochlear HCs in diabetic mice.

The upregulation of Trx expression by SF inhibited DM-induced cochlear HC injury. Treatment with PX12, a Trx inhibitor, reversed the protective effects of SF on cochlear HCs. Trx upregulation by SF inhibited DM-induced cochlear HC injury by downregulating ASK1 and Txnip. In addition, mice overexpressing Trx exhibited delay in diabetes-induced cochlear HC degeneration. The molecular mechanisms underlying otoprotection by Trx overexpression involve regulation of mitochondrial biogenesis, the apoptotic pathway, ER stress, and autophagy. The overexpression of Thioredoxin (Trx) has been found to delay cochlear hair cell degeneration induced by diabetes by regulating various cellular processes. Trx overexpression resulted in increased expression of PGC1α and the levels of Bax were significantly decreased in the Trx overexpressed diabetic group compared to the control diabetic group. Conversely, Bcl-2, an anti-apoptotic protein, was significantly decreased in the control diabetic group but increased in the Trx overexpressed group. Markers of endoplasmic reticulum stress (ASK1, GRP78, and CHOP) were significantly increased in the control diabetic group but decreased in the Trx overexpressed group. The expression of Txnip, a regulator of cellular redox state, was significantly increased in the control diabetic group but reversed by Trx overexpression. The levels of Lc3-OO, a marker of autophagy, were decreased in the control diabetic group but increased in the Trx overexpressed group. The expression of p62, a protein involved in autophagy regulation, was increased in the control group but decreased in the Trx overexpressed group. These findings suggest that Trx overexpression plays a protective role in diabetic-induced cochlear hair cell degeneration by modulating mitochondrial function, apoptosis, ER stress, and autophagy.

Based on the study findings, Trx upregulation could be a potential therapeutic approach for delaying cochlear hair cell degeneration in diabetes. The study emphasized the importance of maintaining mitochondrial function and consuming antioxidants to upregulate Trx expression, preventing diabetic hearing impairment. This study highlighted the association between DM and hearing impairment as well as proposed Trx as a protective factor against DM induced cochlear hair damage.

## Discussion

DM is a multifaceted metabolic disorder known to cause various complications, including hearing impairment. In this systematic review, we analyzed 12 studies that investigated the relationship between DM and SNHL using preclinical animal models. The studies shed light on the impact of high blood glucose levels on hearing function, the molecular and histological changes associated with DM-induced hearing impairment as well as the role of cochlear synaptopathy, and the susceptibility of diabetic animals to noise trauma. We also discussed the utility of animal models in evaluating the efficacy of emerging therapeutic agents in protecting against DM associated HL.

Akcay et al. conducted a study using rat models to assess the effects of blood glucose levels on hearing, utilizing Brainstem Auditory Evoked Potentials (BAEP) and Distortion Product Otoacoustic Emissions (DPOAEs) in a streptozotocin-induced diabetic model [49]. The diabetic group exhibited reduced auditory sensitivity, shown by elevated BAEP thresholds at 8 kHz and 16 kHz, while the high blood glucose (HBG) group only showed delays at 8 kHz. In the diabetic group, the latency of I-V waves was extended at both frequencies, unlike the HBG group, which displayed no significant changes. Moreover, DPOAE values were significantly lowered in the diabetic group, indicating damage to the cochlea and auditory pathways. These findings underscore the critical role of maintaining glycemic control in preventing hearing impairment and the potential detrimental effects of hyperglycemia on auditory function. Future investigations can expand auditory testing in animal models to a broader range of frequencies, to comprehensively assess the spectrum of hearing impairment in DM.

Lee et al. investigated the correlation between T1D and hearing loss (HL) in Akita mice compared to wild-type (WT) mice [50]. The Akita diabetic mouse model exhibited significant elevated glucose levels and insulin sensitivity compared to WT group and at 3 months exhibited increased ABR thresholds at 16 and 32kHz, indicating hearing impairment associated with T1D. Analysis of the Akita group’s cochleae showed significant neuronal reduction, abnormal mitochondria in spinal ganglion neurons, and decreased thickness in the stria vascularis with reduced CD31 expression, indicating vascular stenosis. These changes suggest T1D-related hearing loss may stem from ion imbalance, cochlear endolymph flow issues, and sensory structure degeneration through apoptosis, highlighting mitochondrial dysfunction and cochlear microangiopathy’s role in diabetic hearing loss. These findings were supported by a meta-analysis finding that DM is associated with SNHL [65]. Future research should compare the findings with the Akita mouse model with other T1D models to validate the results and understand if different diabetes models lead to varying degrees or types of hearing impairment.

In another study, diabetic mice exhibited significantly increased hearing thresholds at 32 kHz from as early as 6 weeks, indicating hearing impairment not fully explained by hair cell loss [51]. Despite similar survival of hair cells in the apex and middle cochlear turns, a notable decrease was observed in the basal turn. Further investigation showed a substantial reduction in synaptic ribbons on inner hair cells and a decrease in ABR, suggesting cochlear synaptopathy as a key factor in diabetic hearing impairment. Additionally, histopathological, and Western blot analyses revealed vacuolated mitochondria with disrupted cristae, elevated pro-inflammatory cytokines, and impaired mitochondrial biogenesis and metabolic pathways in diabetic mice cochleae, indicating mitochondria-mediated apoptotic cell death. These findings highlight the need for further research to confirm the role of spiral ganglion neuron loss via mitochondrial apoptosis and underscore the potential of mitochondrial-targeted therapies in treating diabetes-associated SNHL.

Cochlear afferent fibers were explored to determine calcium buffering protein calretinin vulnerability to hyperglycemic insults in a streptozotocin-induced mouse model of diabetes [52]. The study found that hyperglycemic mice had increased damage to their cochlear afferent nerve fibers and a decreased proportion of calretinin-poor cochlear afferent nerve fibers, and a decreased proportion of calretinin-poor cochlear afferent fibers compared to normoglycemic mice. These findings suggest that calretinin might provide an important neuropathy in cochlear afferent fibers and calretinin-poor cochlear afferent fibers might be used as an early cellular marker of SNHL.

A study successfully created both short-term and long-term diabetic rat models, showing that while baseline hearing thresholds were similar between diabetic and control groups, only the control group recovered from hearing threshold shifts after 14 days of noise exposure [53]. These findings suggest that DM may lead to permanent hearing loss post-noise exposure. Interestingly, diabetic animals showed less ribbon synapse damage from noise compared to controls. These results enhance our understanding of how diabetes affects hearing and cochlear synapse changes, underscoring the need for more research into treatments for SNHL in individuals with DM.

Haryuna et al. found that type IV collagen expression in the cochlear lateral wall was significantly lower in diabetic rats compared to controls, indicating the detrimental effects of diabetes on cochlear fibroblasts [55]. This decrease in collagen could be due to the breakdown of extracellular matrix proteins by the plasminogen-plasmin system [66] and the dysregulation of the metalloproteinase system associated with diabetes [67]. However, curcumin treatment in diabetic subjects led to an increase in type IV collagen expression, suggesting its potential in maintaining the cochlear lateral wall’s structural integrity. This aligns with findings on curcumin’s role in stimulating TGF-β1 in fibroblasts [68], highlighting its therapeutic potential in protecting cochlear structure in diabetes. Additional studies are needed to evaluate different dosages of curcumin and their effects on the damage caused by diabetes, as well as to determine safe levels of curcumin consumption. These studies could explore curcumin’s ability to prevent or reduce fibroblastic damage in diabetes mellitus-induced hearing loss. While the initial results are encouraging, ongoing research is necessary to confirm curcumin’s efficacy as a protective and therapeutic agent in treating DM -associated SNHL in humans.

Hong et al.’s study on Huotanquyu granules showed promising results in reducing cochlear damage in diabetic rats [56]. Treatment with these granules increased spiral ganglion cells, reduced basement membrane and stria vascularis thickening, and improved hair cell morphology in the cochlea. These findings indicate Huotanquyu’s potential in preserving cochlear structure and function in diabetes. However, further research is necessary to validate these findings, explore their applicability to humans, determine optimal dosages, and understand Huotanquyu’s exact mechanisms of action.

Xing et al investigated the impact of Asiaticoside, an antioxidant, on cochlear hair cells and hearing loss in SZM-induced diabetic mouse models [57]. Diabetic mice treated with Asiaticoside showed improved hearing and preserved inner and outer hair cells, potentially by reducing AGE/RAGE/Nf-Kb levels. This aligns with human studies showing higher hearing thresholds in diabetics [69]. These results suggest Asiaticoside’s potential as a protective agent against diabetic hearing loss. Future studies should investigate how Asiaticoside interacts with antioxidants and the AGE/RAGE/Nf-Kb pathway in hearing preservation.

Castaneda et al examined the potential of Trigonelline to mitigate diabetic induced auditory damage using a diabetic mouse model [58]. Diabetic mice exhibited higher hearing thresholds compared to non-diabetic controls and treatment with Trigonelline at 10 and 20 mg/kg significantly reduced hearing thresholds. Hearing improvement was accompanied by enhanced cochlear response and increased nerve growth factor (NGF) expression, known for its vital role in the auditory system’s development and function [70]. These results indicate that Trigonelline could protect hearing through nerve growth signaling, presenting a potential treatment for SNHL in diabetes. There is a need to assess the long-term effects of Trigonelline on auditory function and determine whether Trigonelline’s protective effects extend to other diabetic complications, particularly those involving nerve damage.

Another study determined the impact of chlorogenic acid (CA) on hearing impairments in diabetic mice [59]. Mice treated with CA showed notable improvements in hearing, evidenced by reduced hearing thresholds at various frequencies. Additionally, these mice experienced shorter AMLR latencies and greater amplitudes, suggesting CA’s potential in mitigating dysfunction in the central auditory pathways. Morphological studies also indicated CA’s role in repairing OHC damage in the cochlea, possibly due to enhanced microvascular perfusion. These results suggest CA as a potentially effective treatment for both peripheral and central auditory issues caused by DM. However, there is a need to perform dose response studies with CA to determine the optimal concentration that provides maximum therapeutic benefits, which will promote its potential translation from bench to bedside.

Ren et al. demonstrated that elevating Trx levels with sulforaphane reduces DM-related cochlear hair cell damage [60]. Trx, known for its role in reducing apoptosis and supporting cell metabolism [71]., helps mitigate cochlear degeneration in DM by influencing mitochondrial function and reducing ER stress and autophagy. These findings suggest Trx as a promising therapeutic target for preventing hearing loss in diabetes, emphasizing the need for further research into Trx-enhancing treatments and the role of antioxidants in maintaining auditory health in diabetic conditions.

### Limitations

This review has some limitations. Firstly, many of the included studies had short durations, precluding the assessment of long-term effects on the studied modalities or physiology. Short-term studies may not capture the gradual progression of SNHL or other physiological changes that occur in the context of prolonged diabetic states. Additionally, short-term studies may not adequately account for the interaction of diabetes with other age-related changes in hearing. As both diabetes and hearing impairment are more prevalent in older populations, it is essential to understand how these conditions interact over time, which requires long-term investigation. Future investigations with longer durations are warranted to provide a more comprehensive understanding of the enduring effects of DM on hearing impairment with the progression of the disease. Additionally, in investigations involving therapeutic agents, further exploration is needed to determine optimal doses that achieve therapeutic effects while avoiding potential adverse effects in humans. It is important to assess not only the immediate effects of a therapeutic agent but also its long-term safety profile, including any potential cumulative or delayed adverse effects.

## Conclusions and future directions

The studies included in this systematic review offer valuable insights into the intricate connection between DM and hearing impairment utilizing preclinical animal models. Notably, hyperglycemia and blood glucose levels were identified as key factors significantly influencing auditory function. The detailed review of these studies has revealed diverse mechanisms involved in DM-induced hearing impairment. These mechanisms encompass cochlear synaptopathy, cochlear microangiopathy, mitochondrial abnormalities, and apoptosis-mediated cell death in the cochlea.

This review highlighted different therapeutic interventions that are being evaluated utilizing animal models such as Asiaticoside, Trigonelline, Chlorogenic acid, and Huotanquyu granules. These interventions have demonstrated promising outcomes by effectively protecting cochlear hair cells, preserving hearing function, and mitigating the severity of cochlear pathology. Furthermore, the findings underscore the potential application of antioxidants and nerve growth factor signaling agents in offering otoprotection, thereby mitigating the adverse effects of diabetes mellitus (DM) on the auditory system.

Although there have been significant strides in comprehending DM-induced hearing impairment, further long-term studies are necessary to understand the progression of SNHL in diabetic animal models. These studies can provide insights into how hearing loss develops over time in the context of DM. In addition, there is a need to investigate how different DM management strategies such as insulin therapy or dietary changes affect hearing function in animal models. These studies will provide a better understanding of how glycemic control is linked to hearing impairment and auditory dysfunction.

Furthermore, subsequent research efforts should delve into targeted therapies aiming to enhance cochlear mitochondrial function, alleviate inflammation, and modulate apoptosis. Given the association between SNHL and social isolation, dementia as well as cognitive decline in humans [72–80], there is a crucial need for the development of innovative therapeutic modalities that address yet-to-be-discovered molecular mechanisms. Translation of findings from animal models to human studies is essential for further validating these results. The integration of current knowledge and forthcoming outcomes holds the potential for a synergistic approach, ultimately advancing the treatment of DM-associated co-morbidities such as hearing impairment.

## Supporting information

S1 Checklist PRISMA 2020 checklist. (PDF)
